# Design of hydroxy-α-sanshool loaded nanostructured lipid carriers as a potential local anesthetic

**DOI:** 10.1080/10717544.2022.2039808

**Published:** 2022-03-04

**Authors:** Fengming Tan, Lulu Xu, Yanling Liu, Huan Li, Dahan Zhang, Cuiying Qin, Yang Han, Jing Han

**Affiliations:** a Department of Pharmaceutical Engineering, Shenyang Pharmaceutical University, Shenyang, China; b Department Center for Medical Science and Technology, Nation Health Commission of the People’s Republic of China, Beijing, China; c School of Chinese Materia Medica, Shenyang Pharmaceutical University, Shenyang, China; d Faculty of Functional Food and Wine, Shenyang Pharmaceutical University, Shenyang, China

**Keywords:** Hydroxy-α-sanshool, local anesthetic, nanostructured lipid carriers, response surface methodology, voltage gate Na^+^ channel

## Abstract

Hydroxy-α-sanshool (HAS), extracted from *Zanthoxylum piperitum*, is commonly used in oral surgery to relief pain. However, the application of HAS is limited in clinical practice due to its poor stability. This study focuses on the design of a novel nano-formulation delivery system for HAS to improve its stability and local anesthetic effect. Hydroxy-α-sanshool loaded nanostructured lipid carriers (HAS-NLCs) were prepared by melting emulsification and ultra-sonication using monostearate (GMS) and oleic acid (OA) as lipid carriers, and poloxamer-188 (F68) as a stabilizer. Besides, the formulation was optimized by response surface methodology (RSM). Then, the best formulation was characterized for particle size, polydispersity index (PDI), zeta potential, entrapment efficiency (EE%), drug loading (DL%), differential scanning calorimetry (DSC), and morphology (transmission electron microscopy, TEM). The obtained HAS-NLCs were homogeneous, near spherical particles with high DL% capacity. The stability of HAS-NLCs against oxygen, light, and heat was greatly improved over 10.79 times, 3.25 times, and 2.09 times, respectively, compared to free HAS. In addition, HAS-NLCs could exhibit sustained release in 24 h following a double-phase kinetics model *in vitro* release study. Finally, HAS-NLCs had excellent anesthetic effect at low dose in formalin test compared with free HAS and lidocaine, which indicated HAS-NLCs were a potential local anesthesia formulation in practice.

## Introduction

Local anesthetic is widely used to lessen pain in the oral procedure (Boyce et al., 2016) by binding to voltage gated Na^+^ (Nav) channels to block influx of sodium into axons (Dib-Hajj et al., 2009). In fact, local anesthetic is basically divided into amides and ester (Wang et al., 2021), such as lidocaine (Zhao et al., 2018) and benzocaine (Lamey, 2005). Sichuan pepper (*Zanthoxylum piperitum*), also known as ‘toothache trees’, is widely used to treat toothache and rheumatoid arthritis in native cultures such as African, Native American, and Asian. Previous studies have shown that an active alkylamide constituent extracted from *Zanthoxylum piperitum* is hydroxy-α-sanshool (HAS), which causes numbness (Bryant & Mezine, 1999) and analgesia (Sugai et al., 2005) during treatment of toothache. HAS not only could induce tingly numbness by targeting and inhibiting two-pore KCNK channels (KCNK3, KCNK9, and KCNK18) (Bautista et al., 2008), but also could act on distinct somatosensory neuron subtypes to mediate sensitivity to pain by blocking various Nav channels such as Nav1.7 (Tsunozaki et al., 2012, 2013). Among the Nav channels subtypes, HAS has strong inhibitory effect on Nav1.3 and Nav1.7 channels, which predominantly expressed in somatosensory neurons such as dorsal root ganglion (DRG) neurons (Black et al., 1996), Aδ mechanical nociceptors (Michael et al., 2012), etc. Besides, HAS can be absorbed rapidly after oral administration or subcutaneous injection (Iwabu et al., 2010; Munekage et al., 2011; Rong et al., 2016), suggesting that HAS might serve as a potential local anesthetic for oral surgery. However, HAS is extremely unstable against oxygen, light, and heat, which restricts its applications in local anesthetic.

Nowadays, nano-formulation delivery system is often used as a novel strategy for local anesthetic due to the high entrapment efficiency (EE%), good stability, excellent sustained release effect, prolonged analgesia time, reduction in the side effect and systemic toxicity (Moradkhani et al., 2018), such as liposomes (Mana et al., 2013; Wang et al., 2021), microsphere (Liu & Lv, 2014), lipid nanoparticles (Li et al., 2019), and microcrystals (Boedeker et al., 1994). Thus, based on the current nano-formulation technology, it is a demand to develop a new nano-formulated application of HAS to improve its stability and its local anesthetic effect in dentistry and oral surgery.

The aim of this study is the design of hydroxy-α-sanshool loaded nanostructured lipid carriers (HAS-NLCs) as a potential nano-formulation of local anesthetic. Our research prepared HAS-NLCs using melting emulsification and ultra-sonication method. In order to improve the EE% and stability, the proper lipid carriers and stabilizer were carried out in preformulation study; and the nano-formulation was optimized by response surface methodology (RSM). The optimized HAS-NLCs was characterized by particle size, polydispersity index (PDI), zeta potential, EE%, drug loading (DL%), differential scanning calorimetry (DSC), and transmission electron microscopy (TEM), and scanning electron microscopy (SEM). Then, this work attempted to evaluate the stability of HAS in HAS-NLCs against oxygen, light, and heat by the retention rate of HAS. The *in vitro* behavior of HAS-NLCs was fitted and analyzed by drug release kinetics models. Finally, we investigated the local anesthesia effect of HAS-NLCs using formalin test compared with free HAS and lidocaine.

## Materials and methods

### Chemicals and animals

Standard HAS (98% purity) was acquired from Aladdin Corporation (Shanghai, China). Soybean oil was acquired from Aladdin Biochemical Technology Co., Ltd. (Shanghai, China). Oleic acid (OA) was acquired from Hengxing Chemical Reagent Manufacturing (Tianjin, China). Coconut oil and olive oil were acquired from Yien Chemical Technology (Shanghai, China). Caprylic capric triglyceride was acquired from ChemeGen (Shanghai, China). Capryol 90, labrafac lipophile WL 1349, gelucire 50/13, gelucire 44/14, and labrasol was acquired from Gattefosse S.A. (Saint-Priest Cedex, France). Isopropyl myristate (IPM) was acquired from Chengdu Best Reagent Co., Ltd. (Chengdu, China). Glyceryl monostearate (GMS) and stearic acid (SA) were acquired from Bodi Chemical Co., Ltd. (Tianjin, China). Linoleic acid was acquired from Yuanye Biotechnology Co., Ltd. (Shanghai, China). Tween 60 and Tween 80 were acquired from Jinshan Pharmaceutical Co., Ltd. (Guangyuan, China). Poloxamer-188 (F68) was acquired from MAYA Reagent (Jiaxing, China). Methanol (HPLC grade) was acquired from Tianjing Concord Co., Ltd. (Tianjin, China). Distilled water was obtained from Laboratory Ultrapure Water System, acquired from Aquapro International Company (Smyrna, DE).

Male Sprague-Dawley mice (SD, 20 ± 10 g) were acquired from the Center of Laboratory Animal Shenyang Pharmaceutical University (Shenyang, China). The procedure for the experimental animals in this study was followed in concordance with the protocol on animal experiments issued by the Shenyang Pharmaceutical University Ethics Committee.

### HPLC analysis of HAS

The amount of HAS was measured via HPLC (Thermo, Waltham, MA) on a system with a column (Promosil C18, 4.6 × 250 mm, 5 μm, Agela Technologies, Tianjin, China) and an L-2400 UV detector (Hitachi, Shiga, Japan). General operation conditions: the mobile phase was methanol:water (70:30, v/v), the flow rate was 0.8 mL/min, column temperature was 40 °C, and the detector wavelength was 254 nm. The standard curve of HAS was constructed by plotting the concentration of HAS versus the peak area normalization data: *Y* = 23455.897*C* + 23641.225, which presented a good linear relationship in a concentration range of 10.25–80 μg/mL (*R*^2^=0.9997).

### Preformulation study

#### Solubility of HAS in lipids and surfactants

To get particles with great loading capacity, HAS should exhibit high solubility in different ingredients (Gaur et al., 2014). Therefore, it is necessary to study the solubility of drugs in the different lipids and surfactant to determine appropriate ingredients for NLCs.

Screening the proper liquid type carriers, the excess amount of HAS was added into 50 μg of liquid ingredients (soybean oil, OA, coconut oil, olive oil, caprylic capric triglyceride, capryol 90, labrafac lipophile WL 1349, IPM, Tween-60, Tween 80, and labrasol) in 1.5 mL EP tubes (Muchen, Wuhu, China). The EP tubes of liquid ingredients were placed in a TS-110 × 50 water bath oscillator (Shanghai Heheng Instrument, Shanghai, China) at 37 °C and 60 rpm for 12 h. Next, the mixture was taken in a H1850 centrifuge (Cence, Changsha, China) and centrifuged at 12,000 rpm for 5 min. The supernatant fluid was collected, diluted with methanol to 5 mL and analyzed by HPLC.

Moreover, in order to determine the solubility of HAS in solid ingredients, solid ingredients (GMS, SA, gelucire 50/13, gelucire 44/14, and F68) were added into the excess amount of HAS at 200 rpm and kept at 5 °C higher than the melting point of solid ingredient, respectively. Then, the mixture was centrifuged at 10,000 rpm for 10 min. The supernatant fluid was collected, diluted with methanol to 5 mL and analyzed by HPLC.

#### Compatibility experiment

To achieve good stability of NLC, compatibility experiment between different solid lipids and surfactant with good solubility should be investigated. Briefly, a mixture of HAS, solid lipid and liquid lipid was heated and stirred until melted. Then, the surfactant solution was slowly added into the mixture at 1000 rpm for 15 min. Finally, the particle size, EE% and stabilization time of emulsion were evaluated.

### Preparation of HAS-NLCs

HAS-NLCs were prepared by melting emulsification and ultra-sonication method (Schwarz et al., 1994). Briefly, HAS (dissolved in ethanol) was added into the melted lipid (solid lipid and liquid lipid). After completely removing the organic solvent, hot aqueous phase containing surfactant was slowly added in the melted mixture, stirring constantly for 15 min. Then, the obtained emulsion was ultrasonicated using a Scientz-IID probe sonicator (Scientz, Ningbo, China) at 160 W (5 s on, 3 s off) for 20 min and cooled in ice bath for 15 min. Finally, the HAS-NLCs dispersion was lyophilized with glucose (2%, w/w) as cryoprotectant using a scientz-10N freeze dryer (Scientz, Ningbo, China), and stored in a brown airtight container for further characterization (Xia & Kong, 2011; Susa et al., 2021).

### Optimization of HAS-NLCs

A three-factor, three-level Box–Behnken response surface design and data analysis were carried out with Design-Expert 10.0 software. The three factors, including ratio of surfactant to drug (*A*), ratio of total lipid to drug (*B*), and stirring rate (*C*), were taken as independent variables, while the particle size, EE%, and zeta potential were considered as dependent variables. The experimental design of three factors of three levels is presented in Table 1. 2D contour lines and 3D response surface map were generated to determine the influence of independent variables on HAS-NLCs. The significance of each variable is described by *t*-test and *p* value, and the goodness of fit of the model is predicted from *R*^2^ value.

**Table 1.** The Box–Behnken response surface design of three factors and three levels.

Factor	Code	Level		
		–1	0	+1
Ratio of surfactant to drug	A	15:1	45:1	75:1
Ratio of total lipid to drug	B	20:1	23:1	26:1
Stirring rate (r/min)	C	750	900	1050

### Characterization of HAS-NLCs

#### Determination of particle size, PDI, and zeta potential

The average particle size (*z*-average), PDI, and zeta potential (*ζ*) of the HAS-NLCs were measured by dynamic light scattering technique using a Zetasizer Nano ZS^®^ (Malvern Instruments, Malvern, UK). Before measurement, the HAS-NLCs dispersion was diluted by a factor of 10 with ultra-pure water to prevent multiple scattering effects. The measurements were performed in triplicate at 25 °C and the results were expressed as mean ± standard deviation.

#### Entrapment efficiency and drug loading

The EE% was determined by centrifugation method (Lv et al., 2020). Briefly, 500 μL of HAS-NLCs was taken into the ultrafiltration centrifuge tube (0.5 mL, 100 kDa, Millipore, Billerica, MA) and centrifuged at 3000 rpm for 15 min. Then, the filtrate from tubes was collected, diluted with methanol to 5 mL and analyzed by HPLC. Besides, equal volumes of HAS-NLCs were broken, diluted to 5 mL with methanol and analyzed by HPLC. The EE% of HAS-NLCs was calculated by the following equation (Moghddam et al., 2016):

(1) $$\text{EE}\%=\frac{W_{\text{total}}-W_{\text{free}}}{W_{\text{total}}}\times 100\%$$

where EE% is the entrapment efficiency of HAS-NLCs; *W*_total_ is the total amount of HAS and *W*_free_ is the amount of HAS-free in HAS-NLCs; their units are mg.

The recovery experiment of EE% was conducted by standard procedures and elaborated in the supplementary material.

The DL% was measured by HPLC. Ten milligrams of freeze-dried HAS-NLCs powders were thoroughly dissolved with methanol, diluted to 5 mL and determined by HPLC. The DL% of HAS-NLCs was calculated by the following equation (Lakhani et al., 2019):

(2) $$\text{DL}\%=\frac{W_{\text{total}}-W_{\text{free}}}{W}\times 100\%$$

where DL% is drug loading of HAS-NLCs; *W*_total_ is the total amount of HAS and *W*_free_ is the amount of HAS-free in HAS-NLCs; *W* is the weight of freeze-dried HAS-NLCs; their units are mg.

#### Differential scanning calorimetry

The melting and recrystallization behavior of all ingredients were observed by a DSC3 differential scanning calorimeter (Mettler Toledo, Stockholm, Sweden). Briefly, samples were put into the aluminum pans and then placed on the sample platform. The empty aluminum pot, taken as the reference pan, was placed over the reference platform. The pans were heated from 25 °C to 230 °C at a constant rate of 10 °C/min under the environment of nitrogen (20 mL/min).

#### Transmission electron microscopy

The morphological observation of HAS-NLCs was performed by a JEM-2100 TEM (JEOL, Tokyo, Japan). Briefly, the sample was placed on a microscopic carbon-coated grid, negatively stained with 2% phosphotungstic acid and examined at 200 kV.

#### Scanning electron microscopy

SEM was conducted by standard procedures and expounded in the supplementary material.

### *In vitro* release study

*In vitro* release study was performed by dialysis membrane diffusion technique. *In vitro* release of drug was evaluated in PBS (pH 7.4) containing with 0.5% Tween-80 (w/v) as release medium for at 300 rpm and 37 °C for 24 h, using a Franz diffusion cell. A dialysis membrane (10 kDa) was placed between the donor chamber and recipient chamber and then fixed by a stainless steel clamp. Two hundred microliters of sample was withdrawn from receptor chamber at 0, 0.25, 0.5, 1, 2, 3, 4, 6, 8, 18, 20, 22, and 24 h, analyzed by HPLC and replaced immediately by equal volume of fresh and pre-warmed PBS, which provided complete sink conditions for HAS base. The experiments were conducted in triplicates. The data of HAS were fitted by linear and non-linear kinetic models (including the zero order, first order, Higuchi, Hixson–Crowell, Korsmeyer and Peppas, Weibull, and double-phase kinetic model) using Origin software version 9.1 and evaluated using a model-dependent approach.

### Stability study

The stability of HAS in HAS-NLCs was evaluated over time on the basis of the retention rate of HAS, compared with free-HAS, under flowing oxygen, different light conditions and different temperatures (Sheng et al., 2018; Mulrooney et al., 2021). Briefly, HAS-NLCs and free-HAS were transferred into beakers (10 mL), and then kept in a dark place with flowing oxygen at room temperature. For light stability, samples were placed in a ZF-20D UV chamber (Gongyi Yuhua Instrument Co., Ltd., Zhengzhou, China) and a Labonce-TPS high-light stability chamber (Jiangsu Labonce Instrument Co., Ltd., Taizhou, China). Each samples were exposed to the UV light (254 nm) or the high-light (4500 ± 500 Lx) at 15 cm distance from the light source at room temperature. For heat stability, HAS-NLCs and free-HAS were stored in amber sealed vials at 40 °C and 60 °C, respectively. Samples under flowing oxygen, light or thermal treatment were withdrawn at each sampling time, diluted with methanol to 5 mL and analyzed by HPLC.

### Formalin test

#### Sample size and allocation to experimental groups

A total of 80 male SD mice were used for the formalin experiment and were randomly assigned to eight different groups receiving intraperitoneal (IP) injection of saline (as blank control group), lidocaine (as positive control group, 2 mg/g), HAS (1 mg/g, 2 mg/g, and 4 mg/g), and HAS-NLCs (1 mg/g, 2 mg/g, and 4 mg/g) 30 min before formalin injection.

#### Experimental procedure

All drugs were dissolved or prepared in saline. For the formalin injection, 20 μL of 2% formalin was subcutaneously injected into the dorsal surface of right hind paw. Then mice were placed in behavioral arenas (10 cm × 20 cm × 15 cm) for observation. The characteristic spontaneous flinching behavior evoked by formalin injection, including holding, licking, or shaking the injected paw, was quantified by counting the number of flinches after the formalin injection (Ok et al., 2021). Two phases of spontaneous pain-related behavior were observed and divided into phase I (0–15 min) and phase II (16–35 min).

#### Statistical analysis

The time–response data are expressed as the mean ± SD per min. The effects of drug on formalin-evoked flinches were compared with those of saline control groups using one-way analysis of variance with Student’s *t*-test. *p*<.05 was considered statistically significant.

## Results and discussion

### Preformulation study

**Figure 1.**
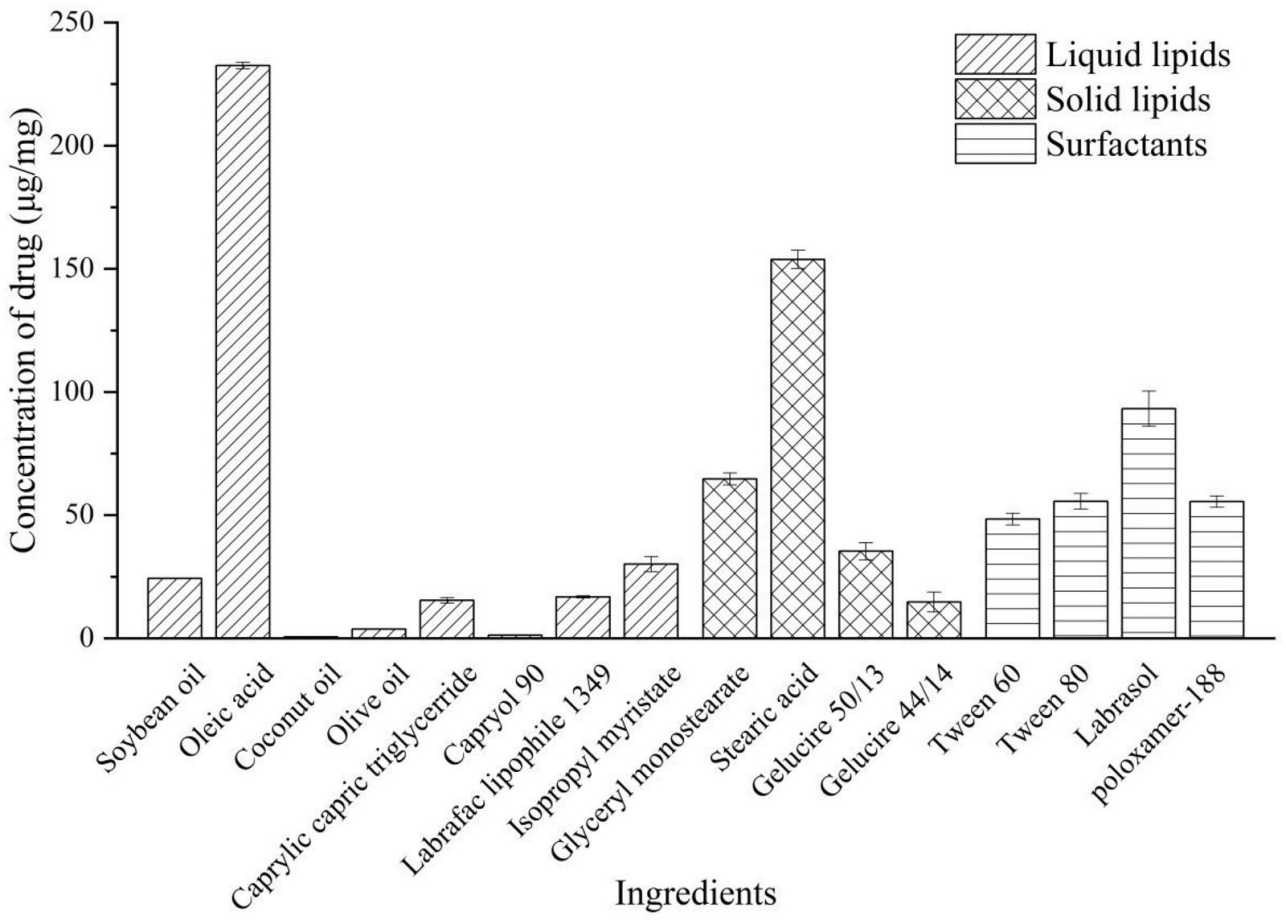
The solubility of HAS in different ingredients.

In Figure 1, it has been shown that HAS reached high solubility in lipids and surfactants, including OA (232.51 ± 1.38 μg/mg), GMS (64.72 ± 2.45 μg/mg), SA (153.85 ± 3.68 μg/mg), tween 80 (55.69 ± 3.17 μg/mg), labrasol (93.22 ± 7.18 μg/mg), and F68 (55.56 ± 2.29 μg/mg). From Table 2, lipid (GMS:OA, 9:1 w/w) and surfactant (F68) might be the most appropriate ingredient system to formulate NLCs with suitable particle size (<200 nm), high loading capacity, and good stability.

**Table 2.** Compatibility experiment result.

Lipid	Proportion (w/w)	Surfactant	Particle size (nm)	EE%	Stable time (h)
SA:OA	9:1	Labrasol	3043.1 ± 36.7	32.51 ± 2.43	<12
SA:OA	9:1	Tween 80	300.48 ± 20.62	67.41 ± 2.72	<24
SA:OA	9:1	F68	289.19 ± 11.62	58.84 ± 3.61	<12
GMS:OA	9:1	Labrasol	1059.4 ± 32.6	48.54 ± 1.34	<12
GMS:OA	9:1	Tween 80	63.32 ± 5.35	89.35 ± 1.58	<24
GMS:OA	9:1	F68	40.21 ± 3.13	96.21 ± 1.75	>48
GMS:OA	8:2	F68	54.97 ± 4.41	90.64 ± 1.43	>48
GMS:OA	7:3	F68	60.48 ± 3.19	89.61 ± 1.56	<48

From the results in Table 2, the addition of different surfactant (F68 < Tween 80 < labrasol) could generally decrease the particle size of the emulsion due to the length of alkyl chain of surfactant (F68 < Tween 80 < labrasol) (Li et al., 2020; Yang et al., 2021). Compared with the formulation of SA and F68, GMS and F68 had better envelopment and stability due to the strong inter-molecular forces (such as hydrogen bonding) between drug and ingredients. As a bio-compatible emulsifier, F68 could maintain the stability of colloidal system by steric hindrance (Anthony et al., 2012; Tsai et al., 2012). Besides, the EE% of NLCs was negatively related to the content of OA. It suggested that solid lipid with a reasonable ratio of liquid lipid could be well mixable and provide high EE% owing to a less ordered lipid matrix (Pardeike et al., 2011).

### Optimization of HAS-NLCs formulation

The response value of the particle size, EE%, and zeta potential of HAS-NLCs was fitted by Design-Expert 10.0 software. After fitting analysis, the quadratic regression model equation was given as follows:

$$\text{Particle size}=\begin{array}{c} 35.21-8.61A+2.53B-1.84C-1.28AB-0.0550AC\\ -0.9425BC+28.67A^{2}+6.25B^{2}-5.61C^{2}\;(R^{2}=0.9213) \end{array}$$

$$\text{EE}\%=\begin{array}{c} 95.41+8.73A+1.71B+0.16C+0.72AB+1.37AC+\\ 1.89BC-10.58A^{2}-0.76B^{2}-0.18C^{2}\;(R^{2}=0.9024) \end{array}$$

$$\text{Zeta potential}=\begin{array}{c} -25.71-4.56A-0.35B-1.81C+2.34AB-1.56AC+\\ 1.69BC+13.44A^{2}+3.25B^{2}+4.42C^{2}\;(R^{2}=0.9341) \end{array}$$

From the analysis of variance of the regression model (in Tables 3–5), the *p* values of the model are 0.0041, 0.0077, 0.0023 < 0.01. The results show that the regression model is extremely significant. The *p* value of the misfit term of the three models is more than .05, indicating that the influence of abnormal factors in the fitting of the regression equation is small, the error between the theoretical value predicted by the regression equation and the actual experimental value in each model is small, and the influence of various factors on different dependent variables can be predicted. The correlation coefficient *R*^2^ >0.9, the degree of fitting is good; indicating that the predicted value of the model is similar to the experimental value with a good correlation. Therefore, the regression equation can be used to determine the optimal conditions for optimizing the preparation of HAS-NLCs.

**Table 3.** Analysis of variance for particle size of HAS-NLCs.

Source	Sum of squares	Df	Mean square	*F* value	*p* Value	Significant
Model	4470.42	9	496.71	9.1	.0041	Significant
*A*	592.54	1	592.54	10.85	.0132	*
*B*	51.11	1	51.11	0.9362	.3655	
*C*	27.2	1	27.2	0.4982	.5031	
*AB*	6.53	1	6.53	0.1196	.7396	
*AC*	0.0121	1	0.0121	0.0002	.9885	
*BC*	3.55	1	3.55	0.0651	.806	
A2	3460.32	1	3460.32	63.39	<.0001	**
B2	179.27	1	179.27	3.28	.1129	
C2	132.63	1	132.63	2.43	.163	
Residual	382.12	7	54.59			
Lack of fit	312.92	3	104.31	6.03	.0576	Not significant
Pure error	69.19	4	17.3			
Cor total	4852.53	16				

*p*>.05 means not significant.

* *p*<.05 means significant.

** *p*<.01 means extremely significant.

**Table 4.** Analysis of variance for entrapment efficiency of HAS-NLCs.

Source	Sum of squares	Df	Mean square	*F* value	*p* Value	Significant
Model	1137.75	9	126.42	7.36	.0077	Significant
*A*	609.37	1	609.37	35.49	.0006	**
*B*	23.32	1	23.32	1.36	.282	
*C*	0.209	1	0.209	0.0122	.9153	
*AB*	2.1	1	2.1	0.1222	.7369	
*AC*	7.48	1	7.48	0.4356	.5303	
*BC*	14.28	1	14.28	0.8319	.392	
*A* ^2^	470.92	1	470.92	27.43	.0012	**
*B* ^2^	2.43	1	2.43	0.1414	.718	
*C* ^2^	0.1292	1	0.1292	0.0075	.9333	
Residual	120.2	7	17.17			
Lack of fit	93.26	3	31.09	4.62	.0868	Not significant
Pure error	26.93	4	6.73			
Cor total	1257.94	16				

*p*>.05 means not significant.

** *p*<.01 means extremely significant.

**Table 5.** Analysis of variance for zeta potential of HAS-NLCs.

Source	Sum of squares	Df	Mean square	*F* value	*p* Value	Significant
Model	1184.74	9	131.64	11.03	.0023	Significant
*A*	166.08	1	166.08	13.92	.0074	**
*B*	0.987	1	0.987	0.0827	.782	
*C*	26.21	1	26.21	2.2	.1819	
*AB*	21.81	1	21.81	1.83	.2185	
*AC*	9.7	1	9.7	0.8131	.3972	
*BC*	11.39	1	11.39	0.9545	.3611	
*A* ^2^	761.07	1	761.07	63.78	<.0001	**
*B* ^2^	44.6	1	44.6	3.74	.0945	
*C* ^2^	82.33	1	82.33	6.9	.0341	*
Residual	83.53	7	11.93			
Lack of fit	68.94	3	22.98	6.3	.0538	Not significant
Pure error	14.59	4	3.65			
Cor total	1268.27	16				

*p*>.05 means not significant.

* *p*<.05 means significant.

** *p*<.01 means extremely significant.

From Table 3, the *p* value of the primary factor *A* is less than .05 and the result is significant; the *p* value of the quadratic terms *A*^2^ is less than .01, which means extremely significant. According to the *F*-value in Table 3, the order factors on particle size were obtained: *A*>*B*>*C*. Besides, the *p* values of the primary factor *A* and the quadratic terms *A*^2^ are less than .01 in Tables 4 and 5, and the results are extremely significant. In Table 5, the *p* value of the quadratic *C*^2^ is .0341 (<.05) and the result is significant. From the *F*-value in Table 4, the order factors on EE% were obtained: *A*>*B*>*C*; meanwhile, the order factors on zeta-potential were gained in Table 5: *A*>*C*>*B*.

**Figure 2.**
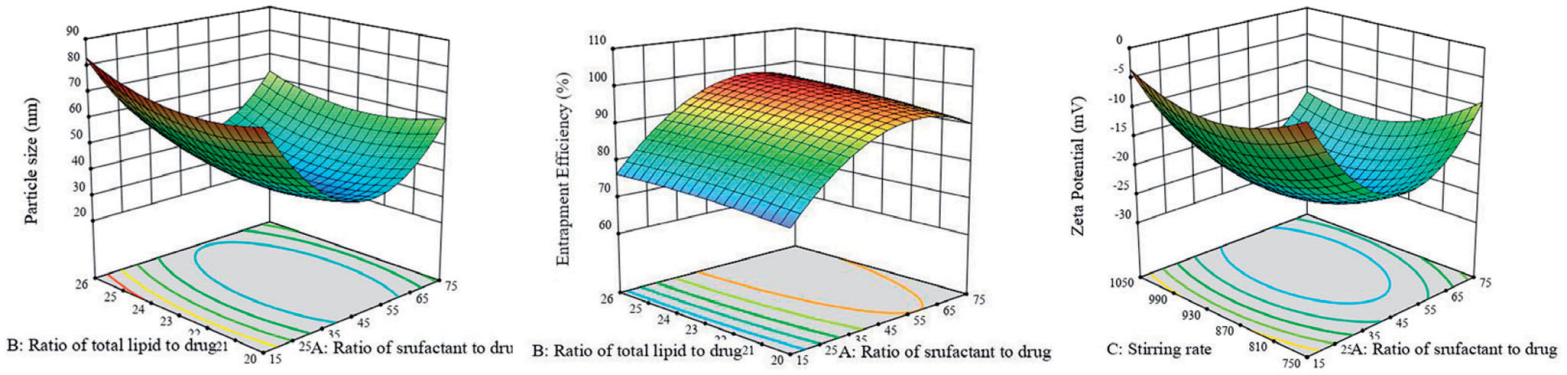
The 3D diagrams and contour maps of different factors on particle size, entrapment efficiency, and zeta potential.

According to the regression analysis, 3D diagrams of the response surface were made for the influence of the three factors on the particle size, EE%, and zeta potential. In Figure 2, there are oval shapes in contour maps, which mean that the interaction between *A* and *B* on particle size and EE%, and the interaction between *A* and *C* on zeta-potential are significant. In addition, the ratio of surfactant to drug (*A*) mostly influenced the response variables. Notably, with an increase in the ratio of surfactant to drug, the particle size and zeta potential of HAS-NLCs decrease at first and then increase, and the EE% increases first and then decreases. It might be attributed that the addition of F68 could efficiently reduce interface tension and prevent drug leaking from lipid matrix, which might result in smaller particle size and higher loading. Small particle size have lager specific surface area, which leads to a reduction in surface charge density. However, overdose of F68 would significantly cause aggregation of particle.

To sum up, the optimal formulation was predicted as follows: the ratio of surfactant to drug was 52:1, the ratio of total lipid to drug was 23:1, and the stirring rate was 990 rpm; the predicted particle size was 34.71 nm, EE% was 97.043% and zeta potential was −25.71 mV. According to the predicted formulation, three parallel experiments were carried out, and the results of particle size, EE%, and zeta potential of HAS-NLCs were gained, which were similar to the predicted value. Also, the adjusted *R*^2^ was more than 0.80, the relative error between the predicted value and the actual value was less than 5%, and the RSD was less than 1%, which judged that the optimum formulation scheme was extremely feasible and provided certain guiding significance for particle.

### Characterization of HAS-NLCs

#### Particle size, polydispersity index, and zeta potential

Particle size, PDI, and zeta potential are considered as important indicators to evaluate NLCs. High zeta potential (higher than |25| mV) might be useful in preserving the stability of particle system by electrostatic repulsion (Singh et al., 2014). From the results, the particle size, PDI, and the zeta potential of HAS-NLCs were 36.61 ± 1.04 nm, 0.131 ± 0.08, and −26.00 ± 0.32 mV, respectively, which demonstrated that HAS-NLCs were homogeneous nanoparticles with physical stability.

#### Entrapment efficiency and drug loading

The EE% and DL% of best formulation were 97.80 ± 0.21% and 3.66 ± 0.12%, respectively, suggesting that this formulation had high DL% capacity. It might be because NLC easily forms an irregular crystal type of lipid core (amorphous type) (Bunjes et al., 2007), which could improve the solubility of HAS and achieve higher EE% (Souto & Muller, 2010; Araujo et al., 2012).

#### Differential scanning calorimetry

**Figure 3.**
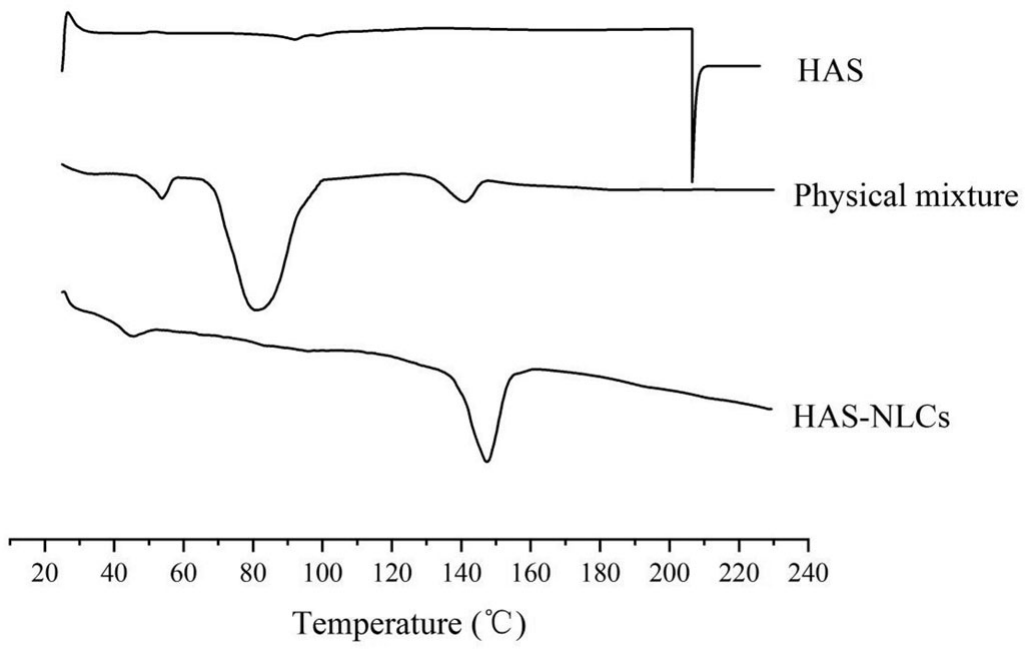
The differential scanning calorimeter thermography of HAS, HAS-NLCs, and physical mixture (without oleic acid).

Differential scanning calorimeter is employed to analyze the physical state and crystallinity of different ingredients with drug (Tatke et al., 2018). In Figure 3, HAS showed a sharp endothermic peak at 206.2 °C, followed by decomposition of drug. Besides, the melting points of F68, GMS, and glucose were basically in the range of 42–68 °C, 65–80 °C, and 140–160 °C, respectively. However, there was not any significant endothermic peak for HAS in physical mixture thermography, which might be attributed that HAS had been completely dissolved in ingredients (Narendar & Kishan, 2016). However, the disappearance of endothermic peaks of all ingredients (except glucose) in HAS-NLCs might indicate the transition of the drug–lipid from crystalline state to amorphous state.

#### Transmission electron microscopy

**Figure 4.**
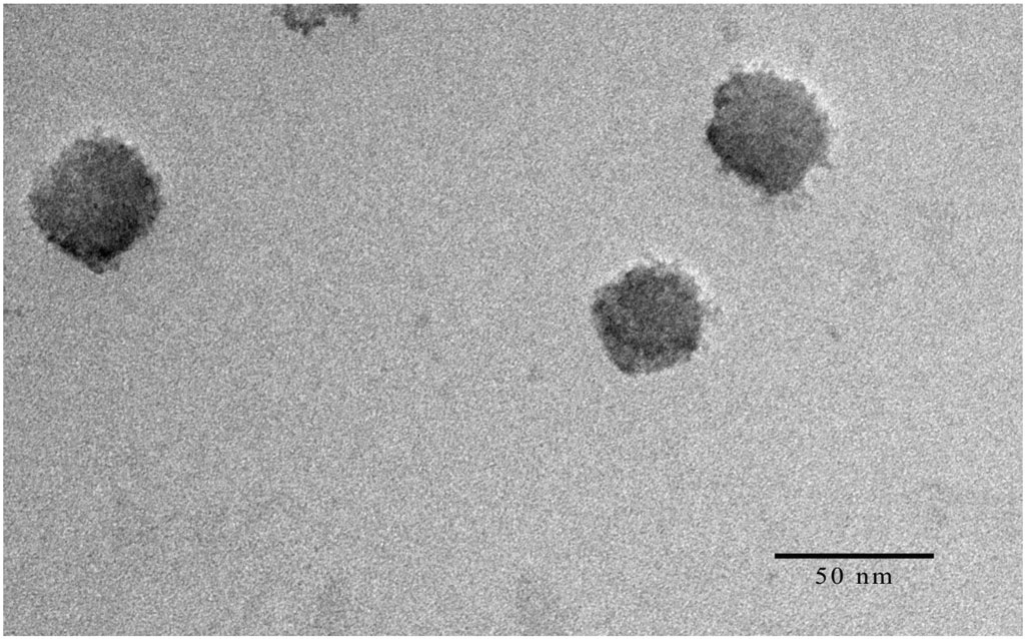
The transmission electron microscopy image of HAS-NLCs.

In Figure 4, the TEM image showed that HAS-NLCs were homogeneously dispersed and nearly spherical nanoparticle. The particle size (35.71 nm) calculated from the TEM image was close to the results measured by a particle size analyzer. It might be confirmed that the HAS loaded nano-formulation carriers were successfully produced.

### *In vitro* release studies

**Figure 5.**
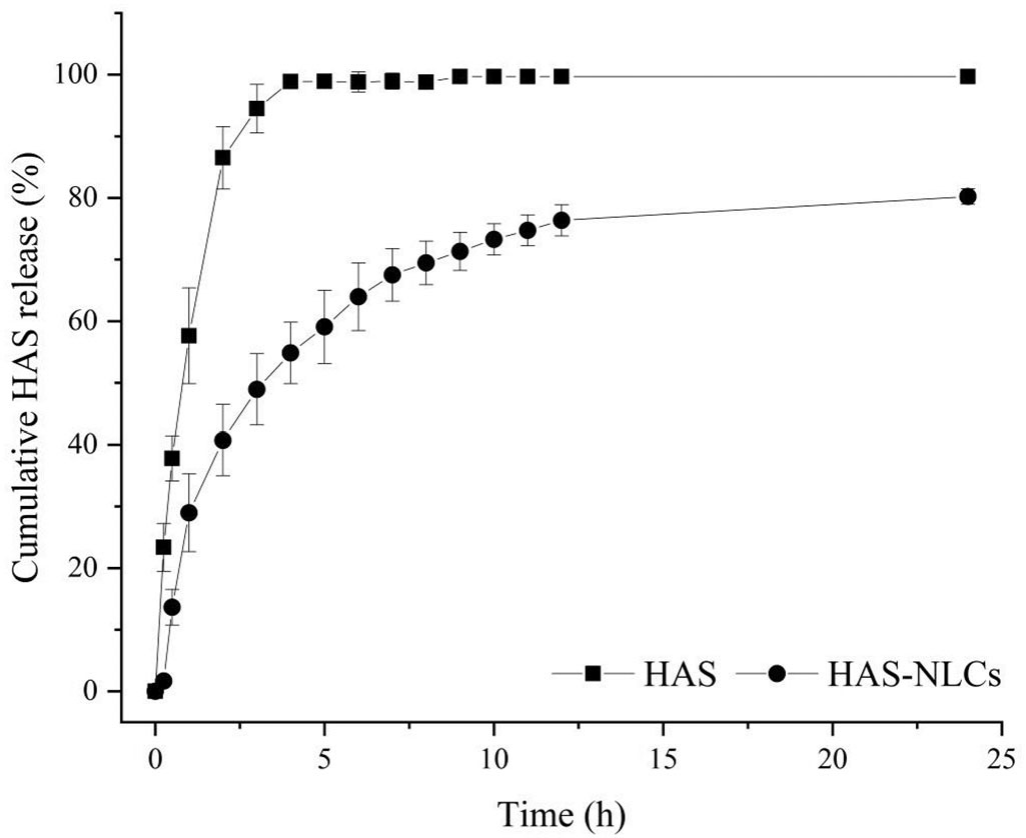
*In vitro* drug release from HAS and HAS-NLCs.

In Figure 5, HAS solution showed 86.49 ± 5.05% of release within 2 h and over 98.87 ± 1.03% after 4 h, whereas HAS-NLCs showed only 40.73 ± 5.75% of HAS release within 2 h and 80.23 ± 1.28% after 24 h, suggesting the extended-release characteristics of HAS-NLCs. Compared with free-HAS, the amount of total drug release decreased 2.12-fold in 2 h. Besides, according to the kinetics model fitting of the drug release profile, it suggested that the drug release was double-phase kinetic model ($y=81.09-54.12e^{-0.19x}-29.84e^{-0.13x},$ *R*^2^=0.9943). Thus, there were two phases in the release of HAS from HAS-NLCs. The impact phase reflected the sudden release effect of nanoparticles within 2 h, probably on account of the rapid diffusion of HAS from the particle surface. The second phase reflected the controlled release of HAS-NLCs after 2 h. The micropore structure of NLC might lead to slow drug diffusion through lipid matrix and enhance the sustained-release effect (Tiwari & Pathak, 2011). It might indicate that HAS-NLCs as local anesthetic could produce controlled-release effect, reduce dosing frequency, and improve patient compliance in clinical practice (Müller et al., 2016; Souto et al., 2020).

### Stability studies

**Figure 6.**
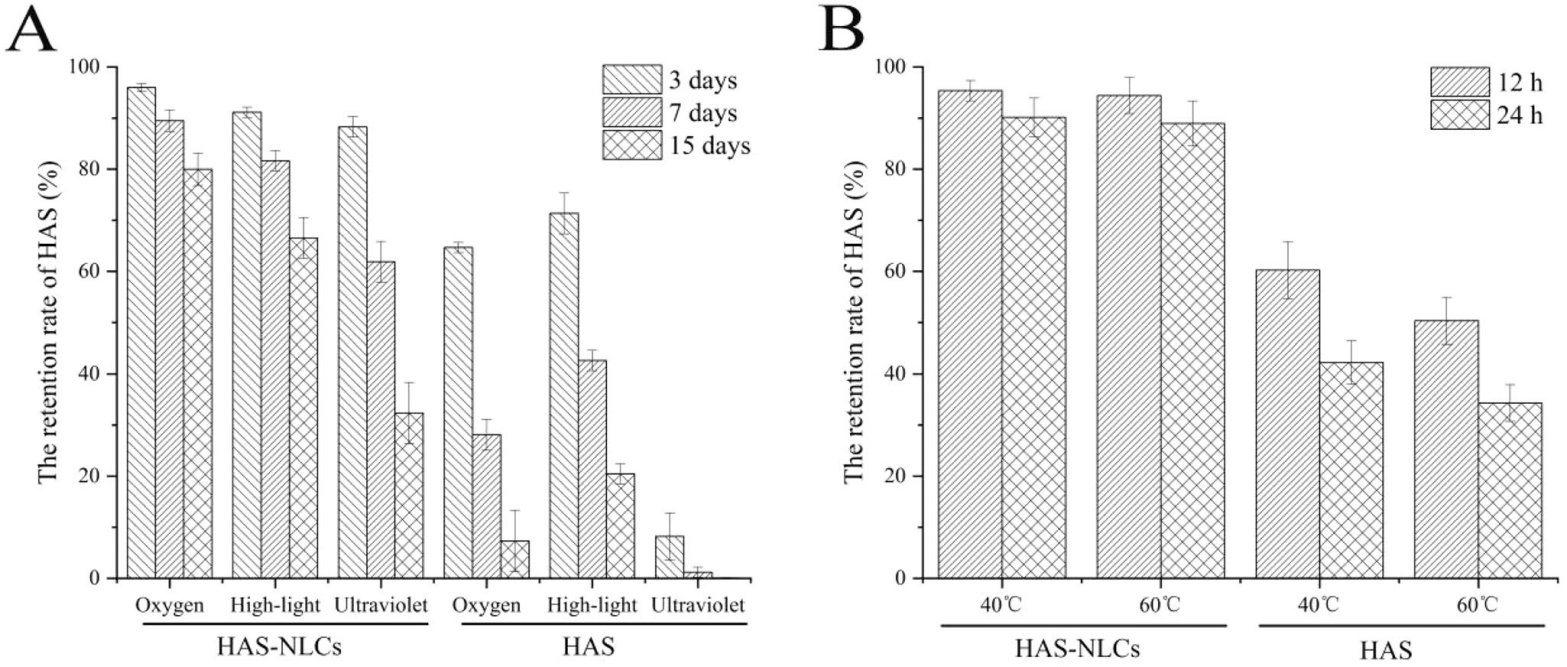
The retention rate of HAS in HAS-NLCs and free HAS under flowing oxygen, high-light, and ultraviolet (A) and at 40 °C and 60 °C (B).

As shown in Figure 6, oxygen, high-light, ultraviolet light, and heat had great impact on HAS, owing to its instability (easy oxidation, isomerization, and hydrolysis) (Yang, 2008). Under oxygen, high-light and ultraviolet light conditions (Figure 6(A)), the retention rate of HAS in HAS-NLCs at day 15 was 10.79 times, 3.25 ,times and 404.38 times higher at day 15 as compared to free HAS, respectively. In Figure 6(B), compared with free drug at 40 °C and 60 °C, the retention rate of HAS in HAS-NLCs was 2.09 times and 2.62 times after 24 h, respectively. It might suggest that HAS-NLCs could effectively maintain the stability of HAS against oxygen, light, and heat, probably because the core–shell structure of NLCs can effectively prevent HAS leaching from lipid matrix and protect HAS from different condition (Nikolić et al., 2011; Liang et al., 2016). Besides, the negative-charged particles with a coverage of F68 might present good stability by electrostatic repulsion and steric hindrance (Khosa et al., 2018).

### Formalin test

The formalin test has been widely used in pain research to study the action mechanism and evaluate the analgesic effect of novel compounds in the preclinical testing (Dubuisson & Dennis, 1977). Two phase of nociceptive behavior is triggered in the formalin test: the phase I is directly related to the stimulation of primary sensory neurons caused by formalin, including nociceptive C-fiber and non-nociceptive Aβ-fiber (Shields et al., 2010); and the phase II is mostly associated with the peripheral and central sensitization induced by inflammatory mediators, such as Aδ-fiber (Rosland et al., 1990; Puig & Sorkin, 1996).

**Figure 7.**
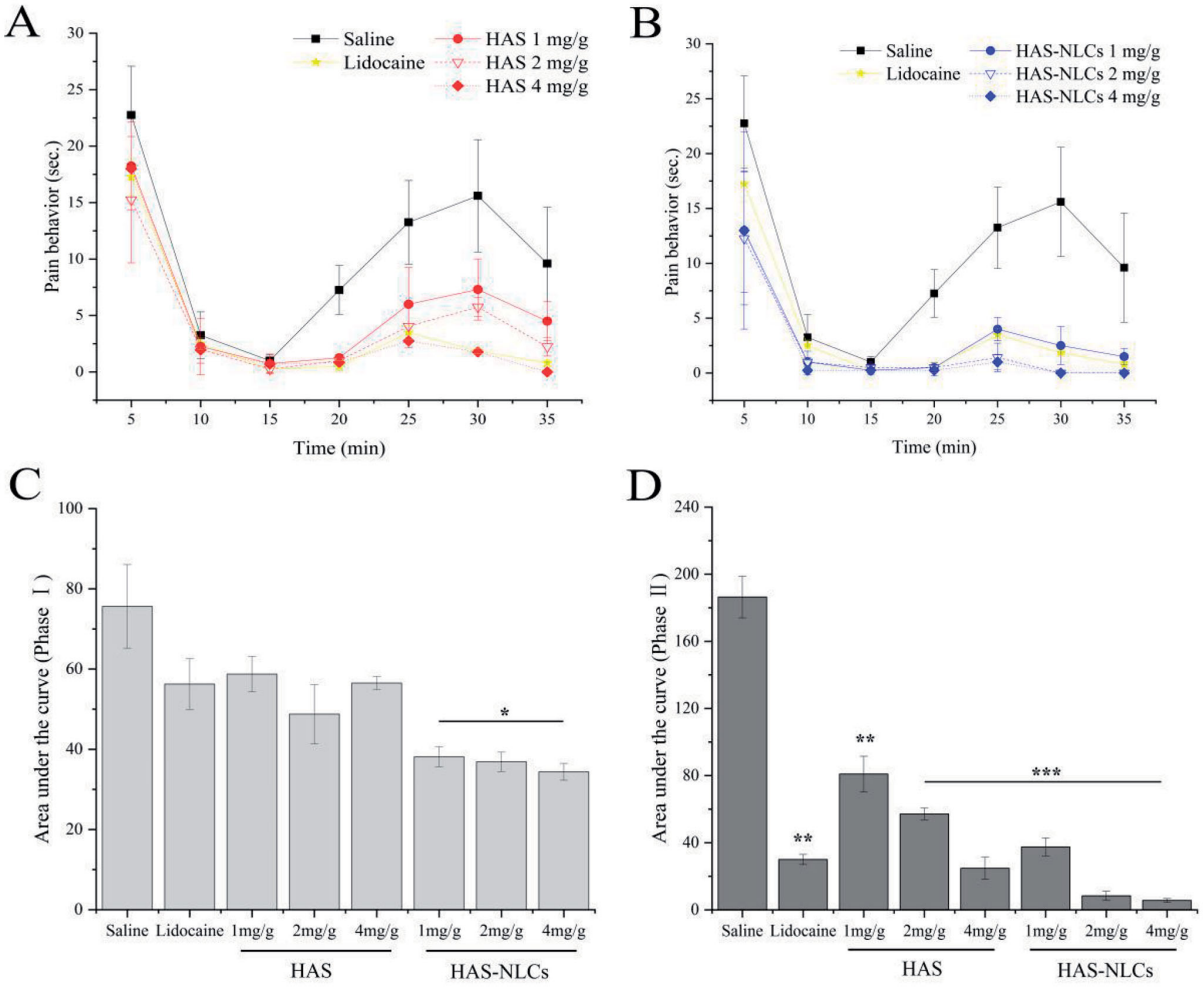
Local anesthetic effect of drug in formalin test. Time–effect curve of HAS and HAS-NLCs on pain behavior response during phase I (A) and phase II (B); plots representing the area under the curve in the phase I (C) and phase II (D) (*p<.05; **p<.01; ***p<.001).

**Figure 8.**
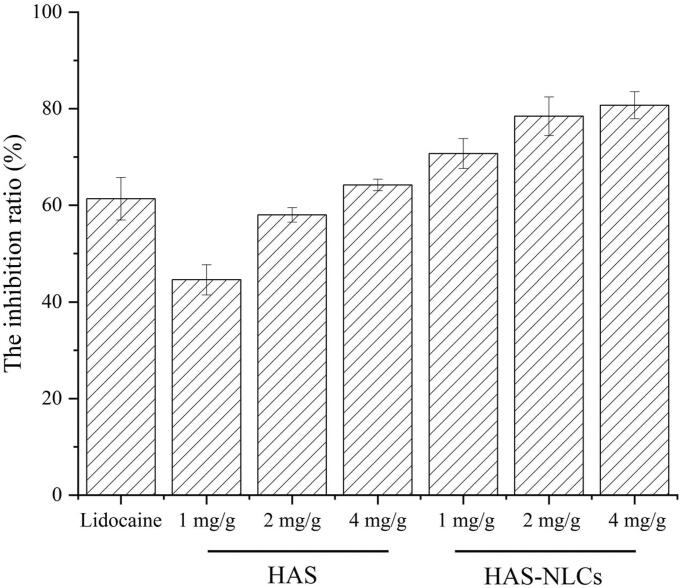
The inhibitory effect of lidocaine, HAS, and HAS-NLCs in formalin test compared to saline.

From Figure 7(A), the HAS groups revealed remarkable pain-easing effect at various phases of the formalin test when compared with the control group. HAS probably could inhibit action potential (AP) firing in somatosensory neurons (such as C-fiber, Aβ-fiber, and Aδ-fiber) by blocking various Nav channel subtypes, thereby effectively reducing pain behavior during the formalin test (Lennertz et al., 2010). Interestingly, compared to the control group in Figure 7(D), for the HAS groups during the phase II (1 mg/g, 2 mg/g, and 4 mg/g), significant reduction of pain behavior was over 55%, 70%, and 85% (*p*<.01, *p*<.001, and *p*<.001), respectively. It might indicate that HAS had better local anesthesia effect during the late phase of the test by selectively silencing Aδ-fiber, which relied on Nav1.3 channel and (or) Nav1.7 channel (Tsunozaki et al., 2012, 2013). In Figure 8, the inhibition ratio of HAS (1 mg/g, 2 mg/g, and 4 mg/g) could reach 44%, 57%, and 64%, respectively, suggesting that the local anesthesia action of HAS was probably in a dose-dependent manner (Mundiya & Woodbine, 2021). Moreover, HAS could achieve similar inhibitory effect on formalin-induced ache model compared with lidocaine at same dose, which verified that HAS could be a promising local anesthetic in practice.

From Figure 7, HAS-NLCs might work rapidly and sustain for longer in formalin test when compared with HAS and lidocaine. During the phase I, the formalin-induced pain behavior of HAS-NLCs (Figure 7(C)) decreased by over 25% and 35%, compared with free HAS and lidocaine groups. It might be because HAS-NLCs with suitable particle size (<50 nm) was composed of bio-compatible lipid and surfactant (as great permeation enhancers), which could be more likely to be transported to the periphery of nociceptive receptors and to block various Nav channels after administration. From Figure 7(D), for the HAS-NLCs groups (1 mg/g, 2 mg/g, and 4 mg/g), the pain behavior during the phase II dramatically decreased by over 54%, 86%, and 77% with a comparison of free HAS, which was attributed that the bio-adhesive lipid in the NLCs might prolong the contact time between particles and neurons (Müller et al., 2011). In addition, the addition of F68 could inhibit the activity of P-glycoprotein to prevent the degradation of particles (Naseri et al., 2015). Compared to free HAS (4 mg/g) and lidocaine (2 mg/g) in Figure 8, the pain-relieving effect of HAS-NLCs (1 mg/g) obviously raised 1.11-fold and 1.14-fold, respectively. It demonstrated that the local anesthesia effect of HAS-NLCs was good at low dose compared to lidocaine, which might decrease complication, increase safety and improve patient’s compliance in clinical practice (Dib-Hajj et al., 2009).

## Conclusions

HAS-NLCs with high EE% and good sustained release effect were successfully developed using nano-formulation technology. Additionally, HAS-NLCs could greatly improve the stability of HAS against oxygen, light and heat (over 10.79 times, 3.25 times, and 2.09 times, respectively). In formalin experiment, HAS-NLCs could produce good local anesthesia effect (1.11-fold and 1.14-fold) at low dose when compared with free HAS and lidocaine. Thus, HAS-NLCs might serve as a safe potential oral local anesthetic with some excellent property as instant absorption, good anesthetic effect, and long action time in practice.

## Supplementary Material

Supplemental Material
