# Supplementary material for: Design of hydroxy-α-sanshool loaded nanostructured lipid carriers as a potential local anesthetic
Source: Drug Deliv. 2022 Mar 4;29(1):743–53. doi: 10.1080/10717544.2022.2039808 (PMC8903781; doi:10.1080/10717544.2022.2039808)
Supplement: Supplemental Material [file IDRD_A_2039808_SM7721.doc]

**Supporting Material**

**Design of hydroxy-α-sanshool loaded nanostructured lipid carriers as** **a potential local anesthesia**

Lulu Xu ^1^,*; Fengming Tan ^1^,*; Yanling Liu ^1^; Huan Li^1^, Dahan Zhang^1^, Cuiying Qin^2^, Yang Han^3^**, Jing Han^4^**

^1^ Department of Pharmaceutical Engineering, Shenyang Pharmaceutical University, No. 103, Wenhua Road, Shenyang 110016, China

^2^ Department Center for Medical Science and Technology, Nation Health Commission of the People’s Republic of China, Beijing, 100044, China

^3^*School of Chinese Materia Medica, Shenyang Pharmaceutical University, No. 103, Wenhua Road, Shenyang 110016, China

^4^*Faculty of Functional Food and Wine, Shenyang Pharmaceutical University, No. 103, Wenhua Road, Shenyang 110016, China

Supplementary Materials and Methods, Figures, Figure Legends

Materials and Methods

**The recovery experiment of entrapment efficiency of HAS**

500 μL of HAS (25 μg/mL, 50 μg/mL and 100 μg/mL) was taken into the ultrafiltration centrifuge tube (0.5 mL, 100 kD) and centrifuged at 3000 rpm for 15 min. Then, the filtrate from tubes were collected, diluted with methanol to 5 mL and analyzed by HPLC. The recovery was found between 99% and 102% (n=9), while the RSD was 0.56%.

500 μL of NLCs and HAS (25 μg/mL, 50 μg/mL and 100 μg/mL) were taken into the ultrafiltration centrifuge tube (0.5 mL, 100 kD) and centrifuged at 3000 rpm for 15 min. Then, the filtrate from tubes were collected, diluted with methanol to 5 mL and analyzed by HPLC. The recovery was found between 99% and 102% (n=9), while the RSD was 0.50%.

**Scanning electron microscopy (SEM)**

The shape and surface morphology of HAS and HAS-NLCs were performed by JSM-6700 SEM (JEOL, Japan). Briefly, the sample coated with gold ions was placed on a carbon strip or coverslip and detected at 20 kV.


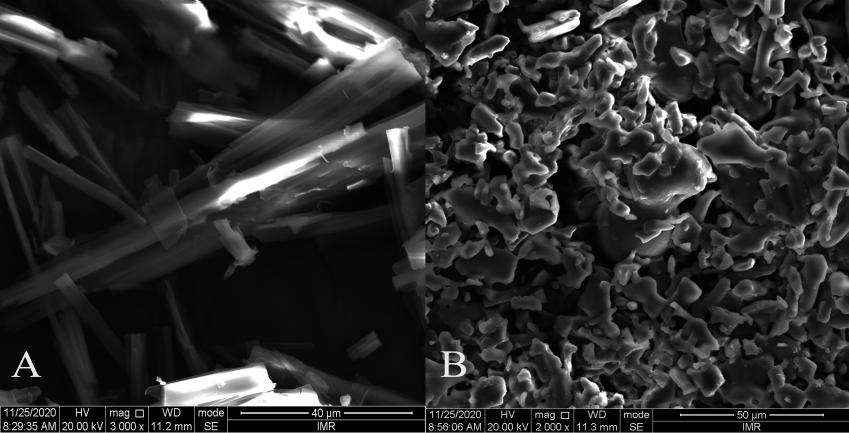


**Figure S1** The scanning electron microscopy image of HAS (A) and HAS-NLCs (B)

In Figure S1, HAS were regularly columnar in shape and HAS-NLCs were nearly spherical without columnar crystals, indicating complete solubility of HAS in NLCs. Besides, there was not any significant agglomeration of NLCs in Figure S1 (B) due to the presence of glucose, which fully covered the surface and effectively inhibited the rupture and conglomeration of particles during lyophilization by water substitution mechanism[[[1]](#endnote-2)].

**References**

1. [?] Y.H. Liao, M.B. Brow, G.P. Martin, Investigation of the stabi-lization of freeze-dried lysozyme and the physical properties of the formulations, Eur. J. Pharm. Biopharm. 58 (1) (2004) 15-24,

   <https://doi.org/10.1016/j.ejpb.2004.03.020>. [↑](#endnote-ref-2)
